# Congenital Afibrinogenemia With Facial Haematoma

**DOI:** 10.7759/cureus.54229

**Published:** 2024-02-15

**Authors:** Mohsin Hassan, Maaz Khan, Moula Ghulam, Nouman Anthony, Mohsin Khan

**Affiliations:** 1 Pediatrics, Lady Reading Hospital, Peshawar, PAK; 2 Internal Medicine, Rehman Medical Institute, Peshawar, PAK; 3 Medicine, Rehman Medical Institute, Peshawar, PAK; 4 General Medicine, Rehman Medical Institue, Peshawar, PAK; 5 Internal Medicine, Rehman Medical Institute, Peshawar , PAK

**Keywords:** simulation medicine, congenital blood disorder, autosomal recessive disorders, bleeding disorder, congenital afibrinogenemia

## Abstract

Congenital afibrinogenemia is a rare inherited blood disorder characterized by a deficiency of fibrinogen, leading to abnormal blood clotting. It is caused by mutations in fibrinogen genes and results in a propensity for bleeding. We present the case of a one-year-old male child with congenital afibrinogenemia who developed a left-sided facial haematoma following a fall from a walker. The child had a history of active bleeding during cannulation and had not undergone circumcision due to the risk of bleeding. This case highlights the need for timely diagnosis and appropriate management of rare bleeding disorders such as congenital afibrinogenemia. Collaboration between different specialties, including haematology and genetic counseling, is crucial for comprehensive care. The rarity of the condition underscores the importance of raising awareness among healthcare professionals. Genetic counseling and family studies are essential for assessing genetic implications and guiding decision-making. Further advancements in diagnostic tests and replacement therapy are needed to improve the management of patients with afibrinogenemia, particularly in regions with a high prevalence of consanguineous marriages.

## Introduction

Afibrinogenemia, also known as congenital afibrinogenemia, is an inherited blood disorder characterized by abnormal blood clotting. It is caused by a deficiency of fibrinogen, a crucial protein (factor I) required for clot formation [1]. The prevalence of afibrinogenemia is estimated to be one in a million [2]. This autosomal recessive disease is caused by mutations in any of the three fibrinogen genes: FGA, FGB, and FGG, which are located on chromosome 4q [3]. These genetic mutations typically result in the premature termination of fibrinogen protein production [4]. The absence of fibrinogen leads to excessive and sometimes uncontrolled bleeding [5]. Symptoms of afibrinogenemia can vary from mild bleeding to life-threatening hemorrhage and are commonly observed in newborns [6]. Haematomas or cerebral hemorrhages brought on by birth trauma, bleeding from the umbilicus, and excessive bleeding during circumcision are some examples of these symptoms [7].

A multi-center study involving 106 cases from the US, Iranian, and Indian registries investigated afibrinogenaemia or hypofibrinogenaemia. The study identified the most prevalent symptoms as mucocutaneous, soft-tissue, joint, genitourinary, traumatic, and surgical bleeding, along with heavy menstrual bleeding (HMB) [8-10]. Pakistan has a high prevalence of inherited bleeding disorders due to the tradition of consanguineous marriages, which leads to the common inheritance of autosomal recessive disorders [11]. Here, we present the case of a one-year-old male child diagnosed with congenital afibrinogenemia who experienced a left-sided facial haematoma following a fall from a walker.

## Case presentation

A one-year-old male child presented to the ED with left-sided facial swelling, bruising below the left eye, and active oral bleeding. These symptoms arose the day after the child fell from a walker, which was two weeks before presentation. The child has a history of previous active bleeding during cannulation and, as a precaution against the risk of bleeding, has not been circumcised. Despite being advised by a local physician to undergo further investigation and medical evaluation, the child did not receive any previous follow-up. He was born full-term via C-section without complications and is the second of two siblings. There is a family history of Wilson's disease, with one affected uncle.

On examination, the child displayed left-sided facial swelling, active oral bleeding, bruising under the left eye, fever, a well-hydrated status, a soft, non-tender abdomen, and equal bilateral air entry on chest auscultation. Initial laboratory findings showed a normal complete blood count but abnormal coagulation parameters. The coagulation profile indicated significantly elevated prothrombin time (PT) (>90.0 seconds), international normalized ratio (INR) (>8.5 seconds), and activated partial thromboplastin time (APTT) (>120.0 seconds).

After the administration of five units of fresh frozen plasma (FFP), the hemoglobin level dropped to 7.3 g/dL, and the red blood cell count decreased to 2.88 x 10^12/L, indicating ongoing blood loss. Additionally, the platelet count decreased to 195 x 10^9/L. The coagulation profile showed an improvement in PT (14.9 seconds), INR (1.37 seconds), and APTT (25.5 seconds). Bleeding time was normal (06.50 minutes), but clotting time was prolonged (>30.00 minutes), suggesting impaired clot formation. These findings indicated the need for further investigation and management of the underlying coagulation disorder.

**Figure 1:**
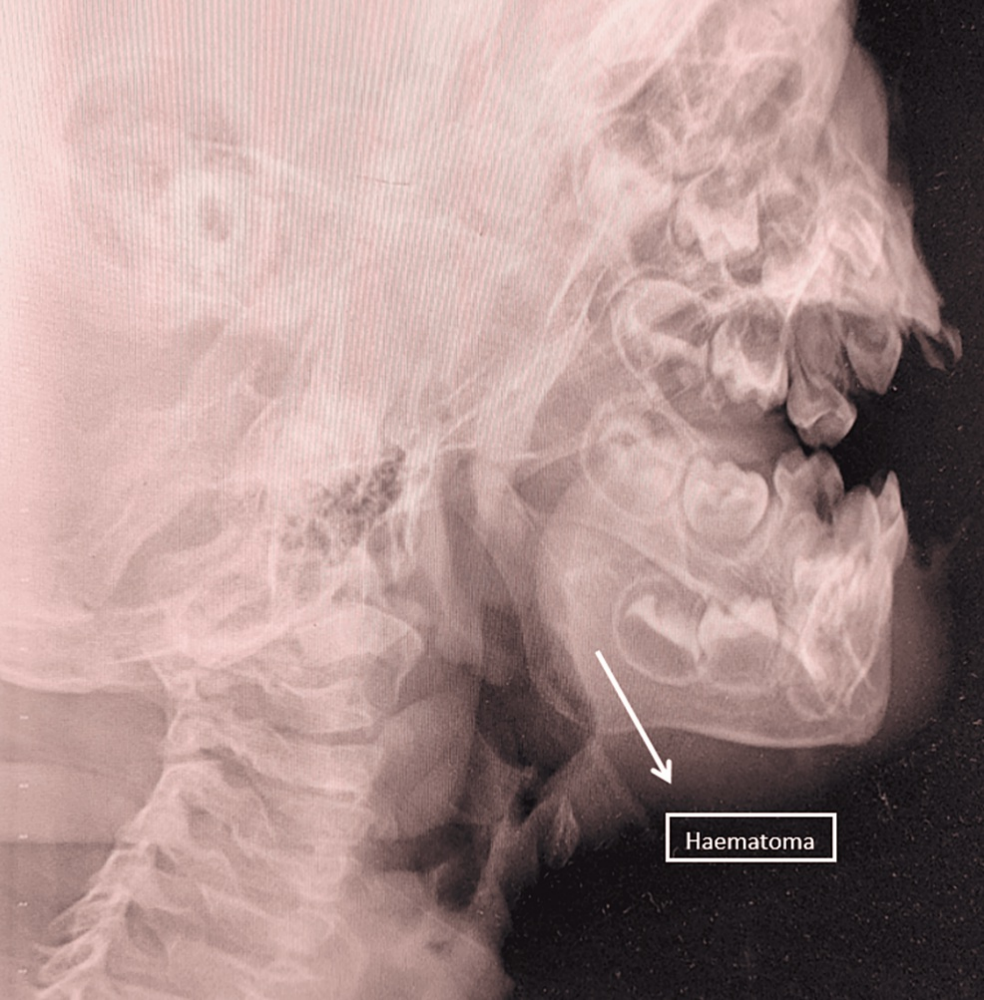
Lateral view of the neck and face X-ray reveals a haematoma

**Figure 2:**
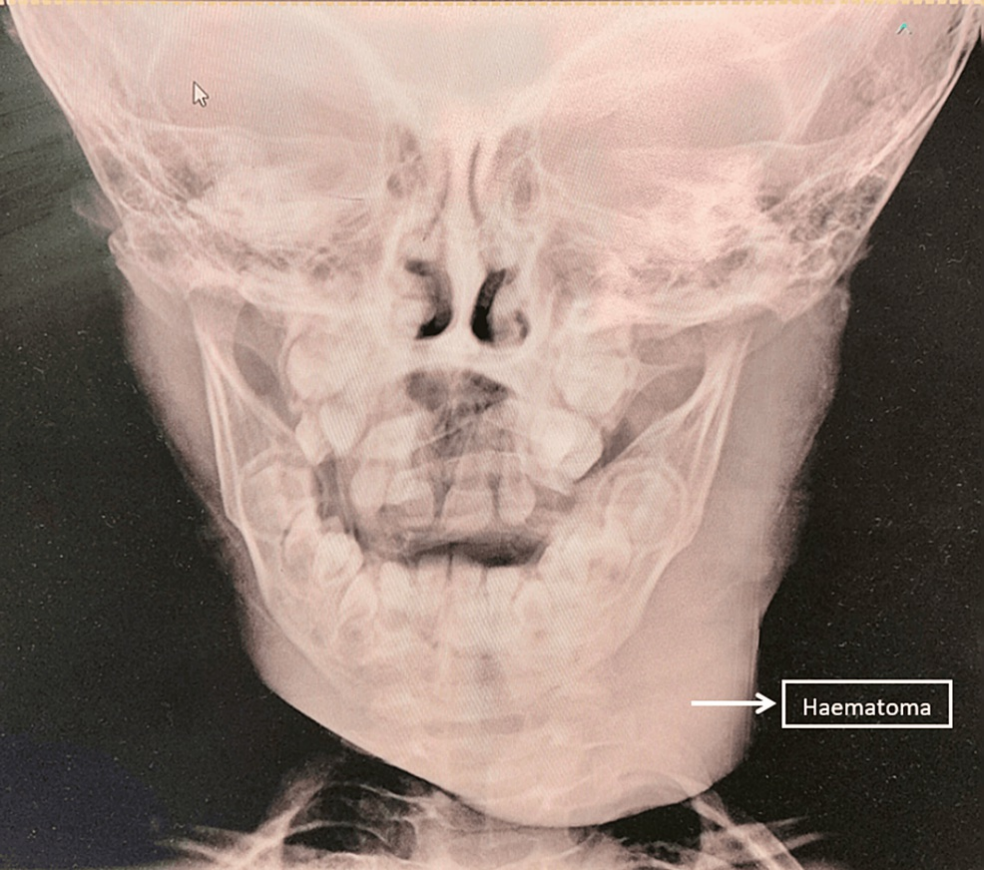
Haematoma seen on the anterior-posterior view of the neck and face X-ray

Additional coagulation tests demonstrated prolonged thrombin time (TT) >60 seconds (normal range 12 to 19 seconds) and decreased plasma fibrinogen levels (96.3 mg/dl), with the normal range being 200 mg/dl to 400 mg/dl, indicating hypofibrinogenemia or afibrinogenemia. An X-ray of the face and neck showed a haematoma on the left side of the face (Figures 1-2).

Ultrasound confirmed the presence of a haematoma on the left side of the patient's face, consistent with the history of the fall. The initial focus was on stabilizing the child's condition by administering intravenous fluids for hydration and Transamine (tranexamic acid) injections to control bleeding. Fresh frozen plasma transfusions were given to address coagulation abnormalities and provide necessary clotting factors. Consultation with a maxillofacial surgeon determined that surgical excision of the haematoma was not necessary. A consultation with a haematologist and hemostasis specialist was also sought to ensure optimal management. Their expertise in fibrinogen replacement therapy guided the treatment approach for coagulation disorders. Genetic counseling and family studies were emphasized to assess potential genetic implications, considering the family history of Wilson's disease. This aimed to provide information, evaluate inheritance risks, and guide decision-making, particularly for individuals with extensive family histories or considering pregnancy. By implementing these multidisciplinary interventions, the treatment plan aimed to stabilize the child's condition, address underlying coagulation abnormalities, and provide long-term management support with genetic considerations.

## Discussion

To the best of our knowledge, this is the first case reported of a patient with congenital afibrinogenemia in the Khyber Pakhtunkhwa province of Pakistan. The clinical presentation of this case aligns with the typical symptoms observed in individuals with afibrinogenemia [12]. The patient exhibited left-sided facial swelling, bruising below the left eye, and active oral bleeding. These symptoms are consistent with the absence of fibrinogen, leading to impaired clot formation and a propensity for bleeding. In patients with afibrinogenemia, bleeding episodes are usually mild, and, in many cases, no spontaneous clinical bleeding is present; bleeding may occur following trauma or surgery [13].

In cases of severe hemorrhages in patients diagnosed with afibrinogenemia, several treatment options are available, such as fibrinogen concentrates, cryoprecipitate, and FFP. Among these options, fibrinogen concentrates are generally regarded as first-line therapy due to their targeted efficacy [2]. However, in our specific setting, fibrinogen concentrates are not readily available, leading us to rely on FFP as the primary choice to manage bleeding episodes effectively. A recommended fibrinogen level for on-demand treatment is typically between 0.5 and 1.0 g/L, varying based on the specific clinical scenario [14].

Genetic counseling and family studies are important components of the management plan for congenital afibrinogenemia [13]. In this case, the family history of Wilson's disease was noted, emphasizing the need for genetic evaluation and counseling to assess potential genetic implications and guide decision-making. Understanding the inheritance risks associated with afibrinogenemia can assist individuals with extensive family histories or those considering pregnancy [15].

This case report underscores the challenges of diagnosing and managing rare bleeding disorders such as congenital afibrinogenemia. Due to the condition's rarity, awareness among healthcare professionals is crucial to ensuring timely diagnosis and appropriate management. We therefore conclude that collaboration and coordination between different specialties, such as haematology and genetic counseling, are essential to providing comprehensive care to patients with afibrinogenemia.

## Conclusions

Children with afibrinogenemia commonly experience spontaneous bleeding in the mouth, nose, and gastrointestinal tract. Due to the inability to form blood clots, minor injuries can result in excessive bleeding or bruising, leading to severe anemia. Supportive treatment is necessary to prevent severe hemorrhage.

Despite being a rare condition, afibrinogenemia might become more prevalent in the future due to consanguineous marriages in our culture. A center for hemophilia should be accessible for patients with afibrinogenemia. The advancement of safer replacement therapies and the creation of new tests to identify patients at higher risk will lead to future improvements in the care of these patients.
